# Cost-Effectiveness of Apixaban Compared with Warfarin for Stroke Prevention in Atrial Fibrillation

**DOI:** 10.1371/journal.pone.0047473

**Published:** 2012-10-09

**Authors:** Soyon Lee, Rachel Mullin, Jon Blazawski, Craig I. Coleman

**Affiliations:** 1 School of Pharmacy, University of Connecticut, Storrs, Connecticut, United States of America; 2 Hartford Hospital, Hartford, Connecticut, United States of America; University of Queensland, Australia

## Abstract

**Background:**

Apixaban was shown to be superior to adjusted-dose warfarin in preventing stroke or systemic embolism in patients with atrial fibrillation (AF) and at least one additional risk factor for stroke, and associated with reduced rates of hemorrhage. We sought to determine the cost-effectiveness of using apixaban for stroke prevention.

**Methods:**

Based on the results from the Apixaban Versus Warfarin in Patients with Atrial Fibrillation (ARISTOTLE) trial and other published studies, we constructed a Markov model to evaluate the cost-effectiveness of apixaban versus warfarin from the Medicare perspective. The base-case analysis assumed a cohort of 65-year-old patients with a CHADS_2_ score of 2.1 and no contraindication to oral anticoagulation. We utilized a 2-week cycle length and a lifetime time horizon. Outcome measures included costs in 2012 US$, quality-adjusted life-years (QALYs), life years saved and incremental cost-effectiveness ratios.

**Results:**

Under base case conditions, quality adjusted life expectancy was 10.69 and 11.16 years for warfarin and apixaban, respectively. Total costs were $94,941 for warfarin and $86,007 for apixaban, demonstrating apixaban to be a dominant economic strategy. Upon one-way sensitivity analysis, these results were sensitive to variability in the drug cost of apixaban and various intracranial hemorrhage related variables. In Monte Carlo simulation, apixaban was a dominant strategy in 57% of 10,000 simulations and cost-effective in 98% at a willingness-to-pay threshold of $50,000 per QALY.

**Conclusions:**

In patients with AF and at least one additional risk factor for stroke and a baseline risk of ICH risk of about 0.8%, treatment with apixaban may be a cost-effective alternative to warfarin.

## Introduction

Atrial fibrillation (AF) is the most common cardiac arrhythmia in the United States (US), affecting about 2.7 million people [1]. It is expected that by 2050, the number of Americans with AF will exceed 12 million. Patients with AF have a 4 to 5-fold increased risk of ischemic stroke, which contributes to significant increases in morbidity and mortality [1], [2].

Warfarin has been shown to prevent up to 64% of strokes in patients AF; however, despite recommendations for its use by consensus guidelines (Class I; Level of Evidence A) [2], warfarin is prescribed in only about half of eligible AF patients [3]. Studies suggest warfarin under-prescribing is a result of prescriber concerns about the increased risk of hemorrhagic stroke and intracranial hemorrhage (ICH), interactions with food and other drugs, the need for and inadequate compliance with international normalized ratio (INR) monitoring and patients' desire to avoid warfarin therapy [4]–[6].

The Apixaban versus Warfarin in Patients with Atrial Fibrillation (ARISTOTLE) trial compared apixaban with adjusted-dose warfarin for the prevention of stroke or systemic embolism in patients with AF and at least one additional risk factor for stroke. Results of the ARISTOTLE trial revealed that apixaban statistically significantly decreased the risk of stroke and by 21%; driven by a 49% reduction in hemorrhagic stroke (p<0.001). In addition, apixaban was associated with a 31% reduced rate of major bleeding (p<0.001), a 38% reduction in ICH (p<0.001) and an 11% reduction in all-cause mortality (p = 0.047) [7]. Based upon the results of ARISTOTLE, apixaban 5 mg twice daily is currently recommended as “a relatively safe and efficacious alternative to warfarin in patients with nonvalvular AF deemed appropriate for vitamin K antagonist therapy who have at least 1 additional risk factor and no more than 1 of the following characteristics: Age >80 years, weight <60 kg, or serum creatinine >1.5 mg/dL, (*Class I; Level of Evidence B*.”[2].

Although apixaban has been deemed a reasonable therapeutic alternative to warfarin, its adoption, usage, and clinical application will heavily depend on its perceived economic value. While assessing the cost of a new therapy based purely on its acquisition costs may provide some insight, the costs or savings associated with such therapies often extend far beyond these baseline figures, especially considering anticoagulation therapy is warranted for a lifetime in patients with AF. Whether the reduction is hemorrhagic stroke and intracranial bleeding with apixaban and the absence of a need for anticoagulation monitoring offsets some or all of the drug's additional cost is unclear. Therefore, we used decision analytic modeling to estimate the costs, quality-adjusted life years (QALYs), life-years saved (LYS) and cost-effectiveness of apixaban compared to adjusted-dose warfarin for the prevention of stroke in patients with AF and at least one additional risk factor for stroke.

## Methods

### The Decision Model Structure and Analysis

**Figure 1:**
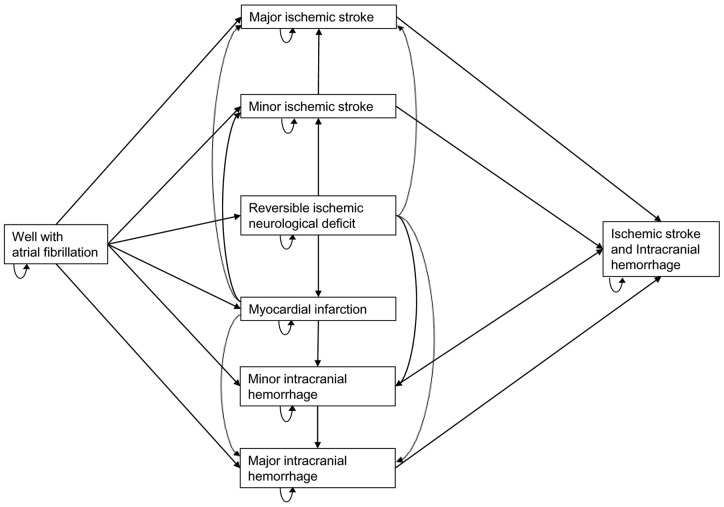
Simple schematic representation of the Markov model. All patients started at age 65 in the “well” with atrial fibrillation health state and then cycled between health states until death occurred or lifetime follow-up period ended (whichever came first). Only certain transitions were allowed and patients could never transition to a more favorable health state. The length of each cycle was 2 weeks and patients could only experience one event of any kind per cycle. Any health state could lead directly to death (not depicted). A second minor ischemic stroke resulted in a major ischemic stroke and that a second major ischemic stroke resulted in death. Temporary health states (e.g., minor bleed and non-fatal extracranial bleed) are not depicted in the figure. The health states were equivalent for apixaban and warfarin, but the probabilities, costs and utilities (quality-of-life) varied with treatment.

We constructed a Markov model to evaluate the cost-effectiveness of two treatment strategies for the prevention of stroke in patients with AF: apixaban 5 mg twice daily and adjusted-dose warfarin with a target INR of 2 to 3. The base-case assumed a hypothetical cohort of 65-year-old patients with AF who had a CHADS_2_ score of 2 and no contraindications to oral anticoagulation. The following health states were modeled: well with AF, reversible ischemic neurologic disease (RIND), ischemic stroke (fatal, major, minor), ICH (fatal, major, minor), extracranial hemorrhage (ECH) (fatal and non-fatal), minor hemorrhage, myocardial infarction (MI) (fatal and non-fatal), and death (**Figure 1**). All patients started in the “well with AF” state, and were allowed to transition from one health state to another based upon defined transition probabilities. Drug-specific transition probabilities were derived predominantly from the ARISTOTLE trial [7]. We incorporated the epidemiological risks of stroke and morbidity and mortality from published studies of anticoagulation identified by means of a systematic search of MEDLINE and a review of the Tufts Cost-effectiveness analysis registry and previous economic models [8]–[12]. We utilized a lifetime time horizon, a cycle length of two weeks, and conducted the analysis from the Medicare perspective [13], [14]. Evaluated outcomes included total treatment cost in 2012 US Dollars, QALYs, LYS and incremental effectiveness ratios (ICERs). Cost-effectiveness was determined as the cost per QALY gained or cost per LYS. All costs and health outcomes were discounted at a rate of 3% per annum [13], [14]. The model was built in TreeAge Pro 2008 (TreeAge Software Inc., Williamstown, Massachusetts).

### Ischemic Stroke Probabilities

We assigned the baseline rate of ischemic stroke on warfarin based on data from ARISTOTLE (1.05%/year, a mean CHADS_2_ score of 2.1±1.1) [7]. (**Table 1**) Based on the ARISTOTLE trial, the rate of ischemic stroke was lower in the apixaban group than in the warfarin group but did not achieve statistical significance [HR = 0.92, 95% confidence interval (CI), 0.74 to 1.13] [7]. For this reason, we conservatively assigned a HR of 1.0 to represent no difference in the risk of ischemic stroke between the two drugs in the base-case analysis. We assumed 28% of ischemic neurologic events would be transient ischemic attacks (TIAs) [15]–[18], and classified ischemic stroke into one of four categories: fatal, moderate-to-severe, mild, or reversible (RIND) [10]. In our model, patients who experienced two minor strokes were subsequently placed in the major stroke health state, and patients who experienced two major strokes progressed to the death health state. Additionally, we assumed that the risk of stroke would increase by 1.4-fold per 10-years of life [9].

**Table 1:** Base-Case Model Variables and Ranges Used in Sensitivity Analysis.

Variable	Base-Case	Range	References
**Costs (2012 US$)**			
Warfarin, 2 weeks (Tablets Only)	15	1–24	42
Cost of INR Laboratory (Per Test)	6	4–10	41
Total Cost of Warfarin and INR Monitoring, 2 weeks	18	3–28	41,42
Apixaban, 2 weeks	95	51–154	43
Aspirin, 2 weeks	0.3	0.07–2.8	42
Event Cost of RIND	6,562	3,500–13,000	33–39
Event Cost of Minor Stroke	9,956	4,500–18,000	33–39
Event Cost of Moderate to Severe Stroke	14,783	11,000–27,500	33–39
Bi-weekly Cost of Minor Stroke	1,232	500–2,000	32–39
Bi-weekly Cost of Moderate to Severe Stroke	2,683	1,000–4,500	32–39
Event Cost of ICH	41,645	16,500–71,000	33–39
Bi-weekly Cost of ICH	2,835	1,000–4,500	32–39
Bi-weekly Cost of Stroke and ICH	3,595	1,600–7,000	32–39
Event Cost of Extracranial Bleed	5,830	2,000–9,000	33–39
Event Cost of Minor Bleed	42	0–200	32–34
Event Cost of MI	20,357	16,500–24,000	39,40
Bi-weekly Cost of MI	152	69–300	39,40
Event Cost of Non-Event Death	5,000	0–10,000	Estimate
Annual Discount Rate (%)	3	0–5	14
**Quality of Life Estimates**			
Healthy on Warfarin	0.987	0.940–1	12.29
Healthy on Apixaban	0.994	0.975–1	7,11,29 (Estimation)
Healthy on Aspirin	0.998	0.994–1	12,29
Major Neurological Event	0.39	0–1	29
Minor Neurological Event	0.75	0–1	29
Disutility of Major Bleed (2 weeks)	−0.16	−0.3 to 0	10–12
Disutility of Minor Bleed (2 days)	−0.16	−0.3 to 0	10–12
MI	0.84	0.5–1	30,31
**Probabilities**			
Baseline Rate of Stroke on Warfarin, %/year (CHADS_2_ Score)	1.05 (2.1)	0.92–1.24	7
HR of Stroke on Apixaban versus Warfarin	1	0.74–1.13	7 (Estimate)
RR of Stroke on Aspirin versus Warfarin	2.08	1.59–2.70	21
RR of Stroke per 10-Years of Life	1.4	N/A	9
Percentage of Strokes with Apixaban or Warfarin that were			
Fatal, %	8.2	8.2–10.1	7,10,23
Major, %	40.2	40.2–41.7	7,10,23
Minor, %	42.5	34.8–42.5	7,10,23
No Residual Deficit, %	9.1	9.1–13.3	7,10,23
Percentage of Strokes with Aspirin that were			
Fatal, %	17.9	10.1–17.9	10
Major, %	30.0	30.0–41.1	10
Minor, %	41.0	34.8–41.0	10
No Residual Deficit, %	11.0	11.0–13.3	10
Baseline Rate of ICH on Warfarin, %/year	0.80	0.63–0.89	7
HR of ICH on Apixaban versus Warfarin	0.42	0.30–0.58	7
RR of ICH per 10-Years of Life	1.97	N/A	20
Percentage of ICH with Apixaban, Warfarin, and Aspirin that were			
Fatal, %	36.4	28.3–45.2	19
Major, %	14.1	9.0–21.4	19
Minor, %	49.5	N/A	19
Baseline Rate of ECH on Warfarin, %/year	3.09	2.59–3.16	7
HR of ECH on Apixaban versus Warfarin	0.69	0.60–0.80	7
Baseline Rate of Clinically Relevant Minor Bleeding on Warfarin, %/year	2.55	2.32–2.80	7 (Estimate)
RR of Clinically Relevant Minor Bleeding on Apixaban	0.69	0.59–0.80	7(Estimate)
RR of Hemorrhage (ICH, ECH, and minor) on Aspirin versus Warfarin	0.87	0.59–0.90	21–23
Baseline Rate of MI on Warfarin, %/year	0.61	0.51–0.76	7
HR of MI on Apixaban	1.0	0.66–1.17	7
RR of MI on Aspirin	1.42	0.84–2.39	25
RR of MI per Decade of Life	1.3	N/A	12,24
RR of Non-Event Death with NVAF	1.3	1.12–1.62	27
RR of Non-Event Death with NVAF and Stroke	2.3	1.3–3.0	28

ECH  =  extracranial hemorrhage; HR  =  hazard ratio; ICH  =  intracranial hemorrhage; INR  =  international normalized ratio; MI  =  myocardial infarction; NA  =  not applicable; NVAF  =  nonvalvular atrial fibrillation; RIND  =  reversible ischemic neurologic event; RR  =  relative risk.

### Hemorrhage Probabilities

We also quantified the baseline rate of hemorrhage (ICH, ECH, or minor hemorrhage) based upon rates observed in patients during the ARISTOTLE trial [7]. Hemorrhages were classified into three categories: ICH, ECH, and the clinically relevant minor hemorrhage (the latter was selected because they would be assumed to result in a patient/clinician encounter) [7]. ICHs were further classified as fatal, major, or minor, and followed the distributions reported by Hylek and colleagues [19]. The ICH rate was also adjusted to account for increasing risk by a factor of 1.97 per 10 years of life [20]. Based on ARISTOTLE, the baseline yearly rate of ECH was set at 3.09% for warfarin, and the ECH rates for those receiving apixaban were derived using the warfarin rate multiplied by the HR of ECH on apixaban [7]. Clinically relevant minor hemorrhage rates for those receiving warfarin was estimated to be 2.55% per year and the HR of clinically relevant minor bleeding on apixaban was 0.69. These values were calculated by subtracting the incidence of the International Society on Thrombosis and Haemostasis (ISTH) major bleeding from the total number of major or clinically relevant nonmajor bleeding events for each drug in the ARISTOTLE trial. We further assumed a major hemorrhage (ICH or ECH) would warrant the discontinuation of apixaban or warfarin and resulted in initiation of aspirin only. We estimated hemorrhage (ICH, ECH, and the clinically relevant minor hemorrhage) risk to be lower for patients started on aspirin as compared to warfarin (RR = 0.87, 95% CI 0.59 to 0.90) [21]–[23].

### Probabilities of MI and Mortality

Our base-case analysis assumed a baseline rate of MI on warfarin as reported in ARISTOTLE, and assumed no difference in the hazard of MI between patients treated with warfarin and rivaroxaban as observed in ARISTOTLE [7]. The risk of MI was increased by 1.3-fold per 10-years of life [12], [24]. The relative risk for MI with aspirin compared with warfarin was estimated at 1.42 from a published meta-analysis by Roskell and colleagues [25].

Age-adjusted mortality rates for non-event (non- ischemic or major hemorrhage) death were derived from the most recent published U.S. Census Bureau estimates and multiplied by a factor of 1.3 to reflect mortality rates in patients with AF [26.27]. We further assumed the risk of non-event death was 2.3 times higher in patients with AF who also developed a stroke [28]. The mortality benefit seen with apixaban in ARISTOTLE was not directly modeled, but rather, was assumed to be the result of decreased clinical events.

### Utility Estimates

We calculated QALYs by multiplying the time spent in each health state by corresponding utilities (quality-of-life) estimates derived from the medical literature (where utility scores range from zero to one; zero representing death and 1 representing perfect health). The inconvenience of INR monitoring in addition to required diet or lifestyle changes were considered when assigning a base case utility value of 0.987 to warfarin [11], [29]. We estimated the utility of apixaban to be higher (0.994) than warfarin as it requires no monitoring, but lower than the utility of aspirin (0.998) because of a potential higher rate of minor bleeding [7], [11], [29].

The utility weight of neurologic events (ischemic stroke or ICH), other major bleeding and MI were derived from the published literature [10]–[12], [29]–[31]. Ischemic stroke (major or minor) and non-fatal ICH had neurologic residual and were assigned a permanent decrement in utility for the remainder of patients' lifetime. Patients who experienced a minor hemorrhage had a temporary disutility for only two days, while patients who experienced a nonfatal ECH had a reduced quality-of-life for one full cycle.

### Costs

Our cost-effectiveness analysis examined direct costs only (inpatient, outpatient and drug); excluding costs due to lost productivity since patients started the model at an age where many are retired from the work force. Healthcare costs were based on data from the Agency of Healthcare Research and Quality's (AHRQ's) Healthcare Cost and Utilization Project (HCUP), the Centers of Medicare and Medicaid Services (CMS), and previously published estimates [32]–[40]. Both one-time costs (transition rewards) and bi-weekly costs were incorporated into the model. One-time costs for RIND, minor stroke, moderate to severe stroke, minor bleed, ECH, ICH, and MI were based on the HCUP costs of a hospitalization for the diagnosis-related group [33]. Two-week costs of care for ICH, stroke and ICH, minor stroke, moderate to severe stroke, and MI were estimated based on the previously published cost estimates from the CMS reimbursement for the diagnosis-related groups [32], [34]–[40]. The cost of a major ECH was estimated by the diagnosis-related group related cost of a gastrointestinal bleed (DRG: 378), since it is typically the most commonly reported major hemorrhage observed in anticoagulation trials, and was the most common ECH in ARISTOTLE. Minor hemorrhage was valued at the cost of an outpatient visit (Current Procedural Terminology code: 99213). The cost of warfarin included the cost of drug plus 14 INR tests per year and the CMS reimbursement for 90-day anticoagulation management [41], [42]. The two-week cost of apixaban ($103) was assumed to be the same as the wholesale acquisition cost (WAC) of rivaroxaban or dabigatran [43]. Costs were expressed in 2012 US Dollars, and inflated when needed using the Medical Care component of the Consumer Price Index [34].

### Sensitivity Analyses

We performed one-way sensitivity analyses of all variables over their plausible ranges along with structural parameters (i.e., time horizon). We derived the ranges of clinical events from the 95% confidence intervals of the event rates reported in the ARISTOTLE trial and from other published literature. To explore the relationship between various risk of ischemic stroke and ICH, we varied these two parameters in a two-way sensitivity analysis. Historical rates of ischemic stroke observed in a national registry of Medicare beneficiaries (baseline rate of stroke on warfarin of 0.61%–5.82% for patients with a CHADS_2_ score of 0–6) were used in this two-way sensitivity analysis [8], [9]. In addition, we conducted a 10,000 iteration Monte Carlo simulation (MCS) to determine the joint uncertainty of model parameters. For each variable in MCS, we assumed a triangle distribution (defined by a likeliest, low and high value) since the true nature of variance for these variables is not well understood and the triangle distribution (when used appropriately) does not violate the requirements of any variable (i.e., costs cannot be less than $0 and probabilities and utilities must lie between 0 and 1). The results of the MCS were plotted on an incremental cost-effectiveness plane.

## Results

Under base-case assumptions, the quality-adjusted life expectancy of 65-year-old AF patients with a CHADS_2_ score of 2.1 was 10.89 and 11.23 years for warfarin and apixaban, respectively. Total costs were $90,225 for warfarin and $87,592 for apixaban, demonstrating apixaban to be a dominant (less costly, more effective) economic strategy compared to warfarin. In analysis not adjusted for utility, the net gain in life-years with apixaban compared to warfarin was 0.20 (11.64 life-years versus 11.44 life-years for apixaban and warfarin, respectively).

### One-way Sensitivity Analyses

**Figure 2:**
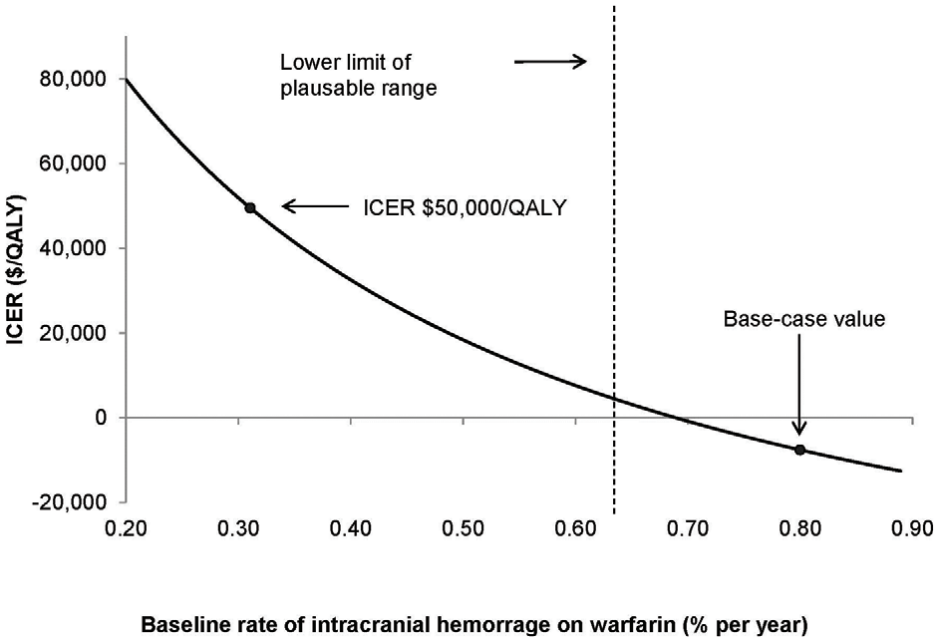
One-way sensitivity analysis of baseline rate of ICH on warfarin. This figure depicts the effect of varying baseline intracranial hemorrhage rates on the ICER. Vertical dotted line demarcates the lower limit of the plausible range (i.e., 0.63% per year).

**Table 2** reports variables for which variations result in a positive ICER (i.e., apixaban is no longer cost-saving but more effective). The base-case results were sensitive to variability in the drug cost of apixaban, treatment costs of warfarin, bi-weekly cost of ICH, the baseline rate of ICH, the relative efficacy of ICH on apixaban compared to warfarin, and time horizon. However, apixaban remained cost-effective (more costly, more effective) using a willingness-to-pay threshold of $50,000 per QALY over all ranges for each variable except time horizon. As the time horizon of the model was varied from 1 to 35 years, the ICER for apixaban became more favorable, eventually falling below the willingness-to-pay thresholds of $50,000 per QALY around year 9 (ICER at 9 years  = $50,392 per QALY). An extended one-way sensitivity analysis on the baseline risk of ICH showed a low baseline risk of ICH (<0.3% per year) was associated with apixaban not being cost-effective (ICER >$50,000 per QALY) (**Figure 2**).

**Table 2:** Results of one-way sensitivity analyses comparing apixaban to adjusted-dose warfarin: parameters for which variations result in positive incremental cost-effectiveness ratios.

Variable	Low range	High range	Threshold value*
Apixaban drug cost, 2 weeks ($)	Dominant	35,583	106
ICH bi-weekly costs, $	26,659	Dominant	2,418
ICH hazard ratio	Dominant	12,049	0.49
Percentage of ICH that are fatal, %	Dominant	1,111	44.0
Baseline rate of ICH on warfarin, % per year	4,899	Dominant	0.70
Cost of warfarin treatment including cost of INR laboratory test, 2 weeks ($)	2,156	Dominant	6.3

ICH  =  intracranial hemorrhage; INR  =  international normalized ratio.

* Value of variable at which apixaban was no longer found to be a dominant economic strategy.

### Two-way Sensitivity Analysis

**Figure 3:**
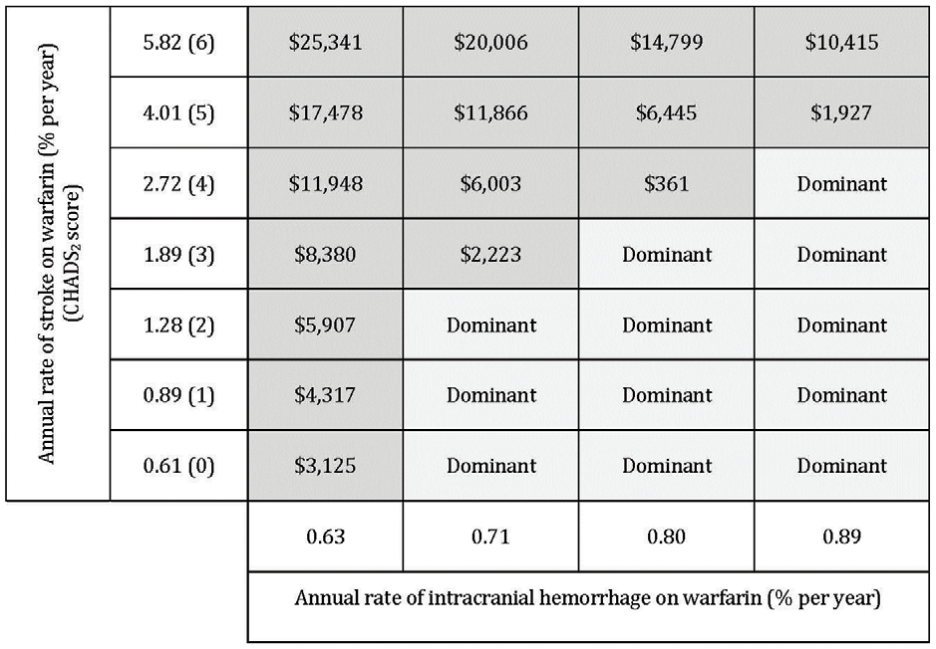
Results of two-way sensitivity analysis. Figure 3 illustrate the different ICERs (cost/QALY) for each combination of values tested for the two parameters (annual rate of intracranial hemorrhage on warfarin and annual rate of stroke on warfarin). Shaded squares represent combinations resulting in positive ICERs and less than $50,000 per QALY gained. ICER  =  incremental cost-effectiveness ratio; QALY  =  quality-adjusted life-years.

Two-way sensitivity analysis of various baseline risks of stroke and ICH demonstrated apixaban is cost-effective when stroke and ICH were varied jointly across plausible ranges (**Figure 3**).

### Monte Carlo Simulation

**Figure 4:**
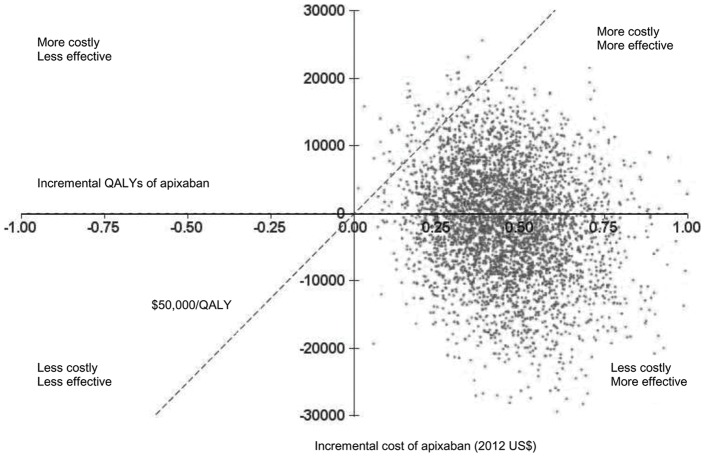
Incremental cost-effectiveness plane. Incremental cost-effectiveness plane showing Monte Carlo estimates of incremental costs and benefits of using apixaban for stroke prevention versus adjusted-dose warfarin. For each one of the 10,000 iterations, values for parameters are randomly selected from their distributions and an ICER is calculated. Points falling above the dotted line have an ICER of >$50,000 per QALY and those falling below the line have an ICER of <$50,000 per QALY. Apixaban was found to be a dominant strategy (less costly, more effective) in 57% of the simulations and cost-effective in 98% of simulations at willingness-to-pay thresholds of $50,000 per QALY. Four thousand of the 10,000 iterations, selected at random, are depicted. ICER  =  incremental cost-effectiveness ratio; QALYs  =  quality-adjusted life-years.

The results of the 10,000 iteration MCS are presented in **Figure 4**. Apixaban was a dominant strategy (less costly, more effective) in 57% of the simulations and cost-effective in 98% of simulations at willingness-to-pay thresholds of $50,000 per QALY.

## Discussion

Our Markov model demonstrates the use of apixaban would modestly decrease the overall cost of treatment ($2,633) and increase quality-adjusted survival (0.34 QALY) among AF patients aged 65 years or older with at least one additional risk factor for stroke and a baseline risk of ICH risk of about 0.8%. These results were however sensitive to variability in a number of parameters estimated in the model. More than half of the time, MCS suggested apixaban would be “cost-saving” (less costly and more efficacious); with the probability of apixaban being cost-effective (assuming a willingness-to-pay threshold of $50,000/QALY) estimated to be over was 98%.

If approved by the Food and Drug Administration (FDA), apixaban will not be the first oral anticoagulant available as an alternative to warfarin for stroke prevention in AF in the United States. Current guidelines for stroke prevention published jointly by the American College of Cardiology Foundation/American Heart Association/Heart Rhythm Society as well as those from the American College of Chest Physicians [44], [45] recommend the oral direct thrombin inhibitor, dabigatran, as an alternative to warfarin for patients who are at moderate to high risk for stroke. [3] Dabigatran has been found to be a cost – effective alternative in this population in a large majority of published economic models [12], [46]–[50]. At present, only a single published Markov model has evaluated the oral factor Xa inhibitor, rivaroxaban's, cost-effectiveness compared to warfarin in this setting [51]. This model which was populated with data from Rivaroxaban Once-daily Oral Direct Factor Xa Inhibition Compared with Vitamin K Antagonism for Prevention of Stroke and Embolism Trial in Atrial Fibrillation (ROCKET-AF) demonstrated rivaroxaban was cost-effective with an ICER of $27,498 per QALY [51], [52]. However, because rivaroxaban was not shown to reduce ischemic stroke in ROCKET-AF (HR, 0.94, 95%CI, 0.75–1.17), the drug's cost-effectiveness was highly predicated on its ability to reduce the rate of ICH (HR, 0.67, 95% CI, 0.47–0.93) [51], [52].

To our knowledge, ours is the first model to assess the cost-effectiveness of apixaban to warfarin. Our model suggests the economics of apixaban are somewhat similar to that of rivaroxaban. Apixaban was also not shown to reduce the risk of ischemic stroke compared to warfarin (HR, 0.92, 95% CI, 0.74–1.13), but did significantly reduce the risk of hemorrhagic stroke by 49% and ICH by 58% [7]. Consequently, it is not surprising that this apixaban model's results were sensitive to many ICH related variables including the baseline rate of ICH, the relative efficacy of apixaban in reducing ICH, and the bi-weekly cost of ICH. Keeping this important finding in mind, clinicians might consider estimating the risk of ICH as one potential means to identify patients who would derive the most benefit from apixaban and thus achieve the most cost-effective use of this new agent. According to our model, apixaban will no longer be a dominant economic strategy if used in patients with an ICH rate <0.7%/year and no longer cost-effective if used in a population with an ICH risk <0.3%/year. Friberg and colleagues recently demonstrated both the HEMORR2-HAGES and HAS-BLED schemes can be used to predict ICH in patients receiving oral anticoagulation (c-statistics, 0.62 and 0.60, respectively) [53]. This report suggested patients with a HEMORR_2_-HAGES score ≥2 or a HAS-BLED score ≥3 would have an ICH rate of 0.7%/year or higher. HEMORR_2_-HAGES and HAS-BLED scores of ≥1 and ≥2, respectively, would correspond to ICH risks of more than 0.3%/year [53].

There are limitations to our analysis that must be considered when interpreting results. First, drug specific transition probabilities were derived solely from ARISTOTLE [7]. Consequently, we had to extrapolate the results of ARISTOTLE, which followed patients for a median of 1.8 years, to the lifetime of a patient with AF [7]. In doing so, we assumed the comparative benefits and harms associated with apixaban would remain constant throughout the entire duration of follow-up. It is possible that rates of adverse events for apixaban or warfarin may vary with a longer follow-up period. Moreover, randomized controlled trial participants and data do not always accurately reflect real-life efficacy and safety because participants may exhibit superior medication management/adherence and receive more comprehensive follow-up. One important example of this is INR control. Patients enrolled in randomized controlled trials have been estimated to spend significantly more time in the therapeutic INR range than patients followed in a community/office setting [54]. Thus, it should not be expected that patients will spend a similar 66% of the time in the therapeutic INR range as observed in ARISTOTLE. A second limitation of our analysis includes our assumption that any major bleeding would result in permanent discontinuation of apixaban or warfarin and initiation of aspirin. It is unclear whether this will always be a clinician's course of action, and would likely be based on individual patient factors. Hopefully with greater use of these newer medications, future models will have more data to refine this assumption. Thirdly, our model did not include other new non-warfarin alternatives (e.g., dabigatran or rivaroxaban). As we have already discussed above, many of the economic models for these newer agents (including our own) are highly sensitive to the relative efficacy and safety estimates used. Thus, evaluating all these alternatives in a single model has the potential to provide misleading results due to the lack of direct head-to-head trial data and the substantial heterogeneity among patients enrolled and methodologies used in the trials. Finally, it should be noted, defining whether an intervention is cost-effective or not must be viewed in context of the willingness-to-pay threshold used [55]. The conclusions of our cost-effectiveness and sensitivity analyses may be interpreted differently, if decision-makers do not prescribe to the $50,000 per QALY threshold used.

### Conclusion

Our analysis suggests that apixaban is likely at minimum cost-effective in patients with AF and at least one additional risk factor for stroke and a baseline risk of ICH risk of about 0.8%. These results are sensitive to several model assumptions; particularly those related to ICH.
